# Barriers and enablers for externally and internally driven implementation processes in healthcare: a qualitative cross-case study

**DOI:** 10.1186/s12913-024-10985-2

**Published:** 2024-04-25

**Authors:** Hilda Bø Lyng, Eline Ree, Torunn Strømme, Terese Johannessen, Ingunn Aase, Berit Ullebust, Line Hurup Thomsen, Elisabeth Holen-Rabbersvik, Lene Schibevaag, David W. Bates, Siri Wiig

**Affiliations:** 1 SHARE - Centre for Resilience in Healthcare, Faculty of Health Sciences, University of Stavanger, Stavanger, N-4036 Norway; 2 Department of Health and Nursing Sciences, Faculty of Health and Sports Science, University of Agder, Kristiansand, N-4604 Norway; 3 Sunnfjord municipality, Førde, N-6802 Norway; 4 Helse Campus Stavanger, University of Stavanger, Stavanger, N-4036 Norway; 5 Kristiansand municipality, Kristiansand, N-4604 Norway; 6 Division of General Internal Medicine, Brigham and Women’s Hospital and Harvard Medical School, Boston, MA USA

**Keywords:** Quality improvement, Implementation, Implementation enablers, Implementation barriers, Nursing homes, Home care services

## Abstract

**Background:**

Quality in healthcare is a subject in need of continuous attention. Quality improvement (QI) programmes with the purpose of increasing service quality are therefore of priority for healthcare leaders and governments. This study explores the implementation process of two different QI programmes, one externally driven implementation and one internally driven, in Norwegian nursing homes and home care services. The aim for the study was to identify enablers and barriers for externally and internally driven implementation processes in nursing homes and homecare services, and furthermore to explore if identified enablers and barriers are different or similar across the different implementation processes.

**Methods:**

This study is based on an exploratory qualitative methodology. The empirical data was collected through the ‘Improving Quality and Safety in Primary Care – Implementing a Leadership Intervention in Nursing Homes and Homecare’ (SAFE-LEAD) project. The SAFE-LEAD project is a multiple case study of two different QI programmes in primary care in Norway. A large externally driven implementation process was supplemented with a tracer project involving an internally driven implementation process to identify differences and similarities. The empirical data was inductively analysed in accordance with grounded theory.

**Results:**

Enablers for both external and internal implementation processes were found to be technology and tools, dedication, and ownership. Other more implementation process specific enablers entailed continuous learning, simulation training, knowledge sharing, perceived relevance, dedication, ownership, technology and tools, a systematic approach and coordination. Only workload was identified as coincident barriers across both externally and internally implementation processes. Implementation process specific barriers included turnover, coping with given responsibilities, staff variety, challenges in coordination, technology and tools, standardizations not aligned with work, extensive documentation, lack of knowledge sharing.

**Conclusion:**

This study provides understanding that some enablers and barriers are present in both externally and internally driven implementation processes, while other are more implementation process specific. Dedication, engagement, technology and tools are coinciding enablers which can be drawn upon in different implementation processes, while workload acted as the main barrier in both externally and internally driven implementation processes. This means that some enablers and barriers can be expected in implementation of QI programmes in nursing homes and home care services, while others require contextual understanding of their setting and work.

**Supplementary Information:**

The online version contains supplementary material available at 10.1186/s12913-024-10985-2.

## Background

Quality in healthcare services is a subject in need of continuous attention [1]. Quality improvement (QI) programmes with the purpose of increasing service quality are therefore of priority for healthcare leaders and governments [2–4]. Nevertheless, despite the number of initiatives and QI programmes aiming to increase quality of care, quality of healthcare has still not improved correspondingly [5].

Programmes for QI usually involve the translation of new knowledge into practice alongside the following implementation and adoption, which is not a straightforward process [6]. Cresswell et al. [7] found implementation processes to be associated with four different contextual factors: technical aspects, social aspects, organizational aspects, and wider socio-political aspects, all influential for the implementation efficiency. This means that to ensure successful implementation, understanding the context specificity, where enablers and barriers for one type of setting not necessarily provide the same type of impact in a different setting, is important [6, 8]. This leads to a need for adapting implementation processes to the situational context [9, 10].

Studies on implementation of QI programmes are still scarce in nursing homes and home care settings. Equally, there is a gap in research concerning similarities and differences of different types of implementation processes, like internally and externally initiated implementation processes. Calls have therefore been raised in literature for future studies to explore contextual aspects in terms of specific types of implementation processes [11–13].

Implementation science has gained increasing focus in health service research, due to the need for understanding the critical role of implementation processes for QI. However, overlapping theories of implementation as well as ambiguous definitions and terminology still challenge the implementation research field [14]. To integrate important contributions, Damschroder et al. [14] in a meta-analysis, proposed five key elements for implementation; Characteristics of the actual intervention/ QI programme, Outer context, Inner context, Individuals involved, and the Implementation process. In a systematic review of barriers and enablers for the implementation of interventions in primary care, Lau et al. [11] presented a conceptual framework displaying influencing key factors for different levels of the implementation process: Intervention/ QI programme factors, Professional factors, Organizational factors, and External context. The understandings provided by Damschroder et al. [14] and Lau et al. [11] display similarities between these frameworks, and their relatedness for this study will be further discussed in terms of the inductive findings of this study in the context of two different implementation processes taking place in nursing homes and homecare services in Norway.

## Aim and research question

This study aims to contribute understanding of two different implementation processes in Norwegian nursing homes and home care services. The first implementation setting (external case) refers to an externally driven implementation process where a researcher group facilitated meetings, material, assignments, monitoring of the process, and follow up. The second implementation setting (internal case) refers to an internally driven implementation process, where the organization itself had decided to implement a specific QI programme, and where the organization was responsible for the full implementation process. The following research questions guided the study:

What type of enablers and barriers are found important for implementation processes in nursing homes and home care services?

And secondly, are identified enablers and barriers different or similar across externally and internally driven implementation processes?

## Method

Based on the exploratory nature of the aim and research questions, we opted for a qualitative methodology [15]. The empirical data were collected through the ‘Improving Quality and Safety in Primary Care – Implementing a Leadership Intervention in Nursing Homes and Homecare’ (SAFE-LEAD) project. The SAFE-LEAD project is a multiple case study of two different QI implementation processes in primary care in Norway [16].

### Contextual setting

A large externally driven QI (leadership guide) implementation study was supplemented with a tracer project [17] involving an internally driven QI implementation to understand the differences and similarities of implementation processes in primary care. Primary care refers to nursing homes and home care services in this study. In Norway, primary care is the responsibility of municipalities. To frame this responsibility there is a regulation stating the requirement of continuously improving quality and patient safety in primary healthcare [18, 19]. This regulation inserts a need for leaders to plan, implement, and evaluate output from QI implementation processes. Leaders are therefore key actors for quality and safety improvements. As such, this study used leaders as informants for developing understanding of QI implementations in a Norwegian primary care setting. Based on the consensus of the importance of context for implementation processes [8, 20–27] this project provides a way of understanding and comparing healthcare implementation processes through two different approaches. However, both implementation processes share similarities, as both are seeking to implement a QI programme, both are within primary care, informants in both cases are leaders, and both are within the Norwegian primary care context. The split of the cases into an externally driven approach and an internally driven approach are not totally binary but represents the main tendency of their approach. However, the external case had to arrange for the homework internally, and the internal case engaged in an externally facilitated day of formal training provided by someone outside their organisation.

### Internal case

Implementation for the internal case took place as an internally driven process in home care. The chosen QI programme was a competence improvement program focusing on observational competence, and included formal teaching of new knowledge, skills training, simulation training of new procedures and measurements, and new practical equipment. Formal teaching involved a day of teaching organized by the county’s Centre for Development of Institutional and Home Care Services (USHT), while the remining was up to the organization to facilitate and organize for (training, simulation, equipment, monitoring, and follow-up) [28]. USHT set the researchers in contact with two different home care districts who had chosen to initiate this specific QI program.

### Data collection

The data collection informing this study was based on individual interviews after the implementation of the competence improvement program, to explore how the implementation process was experienced and evaluated. Only interviews of leaders and professional development nurses (nurses responsible for the professional development within the organization and as such hold an informal leader role) (*n* = 8) were included in the dataset, to ensure credibility in the cross-case comparison, see Table 1. Researcher TS performed all interviews, following a semi- structured interview guide (supplementary file). All interviews were recorded and transcribed verbatim.

**Table 1:** Contextual setting

	External Case	Internal Case
**Setting**	Leaders in nursing homes and home care services	Leaders in home care services
**Study period**	12 months (March/April 2018 - March/April 2019)	2017/2018–2020
**Participants**	Home care 1: District unit, Medium sized municipality, Employees: <100 Home care 2: Rural unit, Small sized municipality, Employees: <100 Nursing home 1: Large city, 7 departments (1short-term, 1 drug care, 3 dementia, 2 long-term), Employees: 200–300 Nursing home 2: Medium city, 1 department including 3 groups (1 dementia, 2 long-term). Employees: <100	Home care A: Large city, 1 of 6 districts in the municipality, 3 groups participated. Leaders and professional nurses. Home care B: District unit, 2 groups participated, Leaders and professional nurses.
**Type of QI programme**	Leadership guide for leaders in elderly care aiming to build competence of quality and safety work	Competence improvement programme for improved competence in recognising and responding to deteriorating frail older patients in primary care.
**Type of implementation process**	1 year implementation process including 3 workshops and 3 “homework” activities for each study site, facilitated by researchers	Multi-component programme including a teaching seminar, a written compendium, a digital learning tool, skills training, simulation-based training, a structured communication tool (ISBAR).
**Recruitment**	Centre for Development of Institutional and Home Care Services (USHT) recruited the study sites in line with the study design (variety of contextual settings)	Centre for Development of Institutional and Home Care Services (USHT) provided contact with units undertaking the specific quality improvement program, these units were requested to take part in the study

### External case

The external case concerned the implementation of a QI programme (SAFE-LEAD) developed by the researcher group aimed at leaders in primary care (nursing homes and home care services) [16, 29]. The QI programme consisted of a leadership guide to support leaders in their QI work. The leadership guide was based on the QUASER hospital guide [6], which was translated and adapted to the Norwegian primary care context [29]. The QI programme included three steps with associated workshops, facilitated by the research group, and with following “homework” for the participants to perform between each workshop. The first workshop aimed at identifying challenges within the organization. “Homework” involved an evaluating and scoring system to formalise challenges of the organization to work on. The second workshop included the development of different objectives for improvement on identified challenges. “Homework” at the second step concerned the development of formalized objectives. At the third workshop, the focus was on the development of action plans in each organization. “Homework” after the third workshop was for leaders to translate action plans into practise. The “homework” sessions provided the participants time to work with the material introduced in the workshop. As such, “homework” was a way to provide ownership to the QI programme by translating the content of the leadership guide to their context specific understanding, setting strategies and objectives for their unit.

### Data collection

The researcher group facilitated the implementation process through workshops focusing on self-diagnosis of the organisation, goal settings, and action plans. The workshops included presentations and knowledge sharing of upcoming tasks, monitoring of progress, discussions, and homework review. The participants were leaders from eight units (4 nursing homes and 4 home care services) in five municipalities, within three Norwegian counties. The researcher group (ER, TJ, IA, BU, LHT, EHR, TS, LS and SW) responsible for the workshops were also performing the interviews. The empirical data used for this study totalled 13 interviews (10 focus group interviews and 3 individual interviews) (*n* = 26) with leaders in all 8 units during a one-year period (April 2018 – March 2019), see Table 1. A semi-structured interview guide was used for the interviews and all interviews were recorded and transcribed verbatim (supplementary file).

### Data analysis

**Fig. 1:**
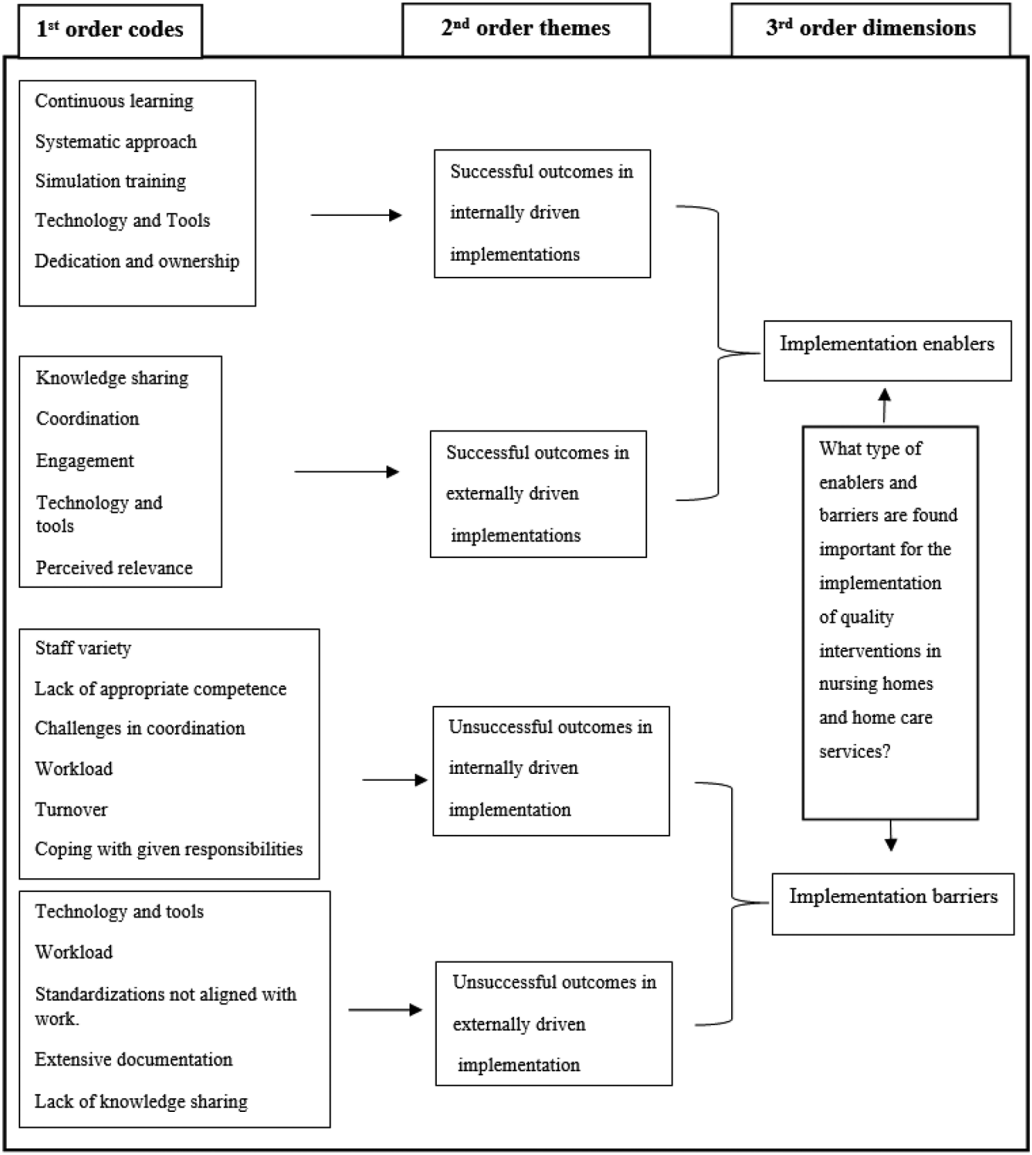
Data structure model based on Gioia et al. [30]

Due to the explorative nature of the research questions, an inductive approach was found most appropriate to explore emerging themes from the empirical data, as existing frameworks describing differences and similarities between internally driven and externally driven implementation processes in older adult care were not identified. This gap in literature informed an inductive approach. The analytical process followed a grounded theory methodology [30, 31] where empirical data from the two cases were first inductively analysed individually, followed by a second step of cross-comparison. Grounded theory is described as a valuable approach for theory development and as such a way to address the theoretical gap described in the above [30]. The NVivo 1.7 software was used to support the analysis and for documentation of findings. Figure 1 illustrates the inductive data structure from the analysis, following the grounded theory framework by Gioia et al. [30]. The data structure includes a first inductive step of identifying 1st order codes emerging directly from the data. These 1st order codes were aggregated into 2nd order themes, and later on into more abstract 3rd order dimensions [30]. As such, 1st order codes display manifest meanings from the dataset. The researcher (HBL) identifying the 1st order codes had not been part of the data collection, workshops, or the QI programme development and was therefore able to keep an inductive approach throughout the analysis. 2nd order themes and 3rd order dimensions were agreed upon by all authors. All authors have expert competence in qualitative inductive research. A summative analysis of the results from the matrix were used to identify enablers and barriers occurring in the dataset.

## Results

The analysis revealed 1845 references which were coded over 284 different inductive 1st order codes emerging from the internal dataset and 3286 references coded at 190 different 1st order codes from the external dataset. A matrix where crosstabulations between successful and unsuccessful outcomes (1st order codes) in each case and the total number of codes provided the association of enablers and barriers displayed in Fig. 1.

Successful outcomes referred to factors, resources, activities, and practices reported to have a positive impact on the implementation process. Unsuccessful outcomes referred to the opposite, where factors, resources, activities, and practices, were reported to have a negative impact on the implementation process. Successful outcomes for the internal case disclosed 122 occurrences and 115 occurrences for the external case. Unsuccessful outcomes for the internal case totalled 110 occurrences and 94 occurrences for the external case.

**Fig. 2:**
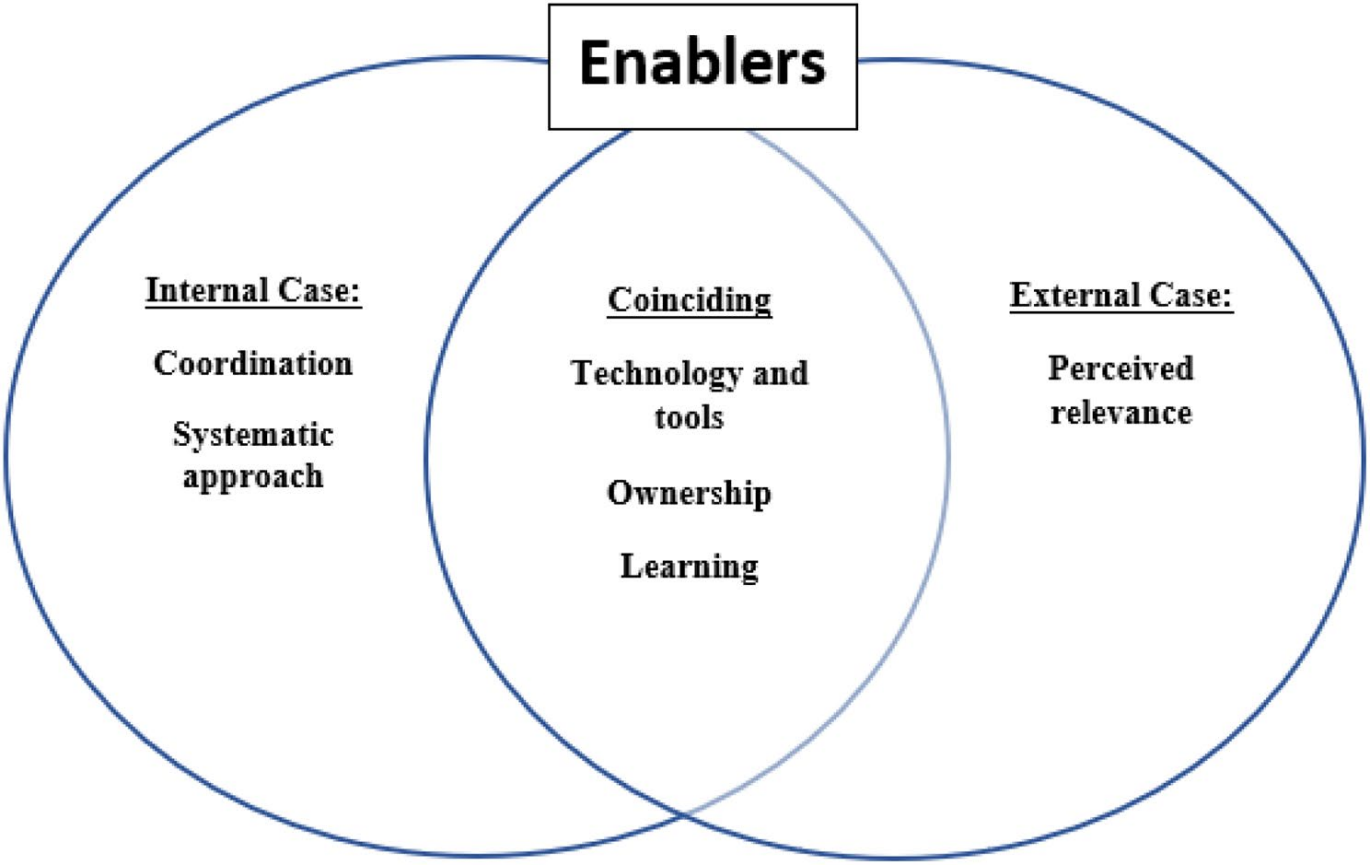
Enabler’s relatedness to externally and internally driven implementation processes. Learning includes both the continuous learning and knowledge transfer enablers

**Fig. 3:**
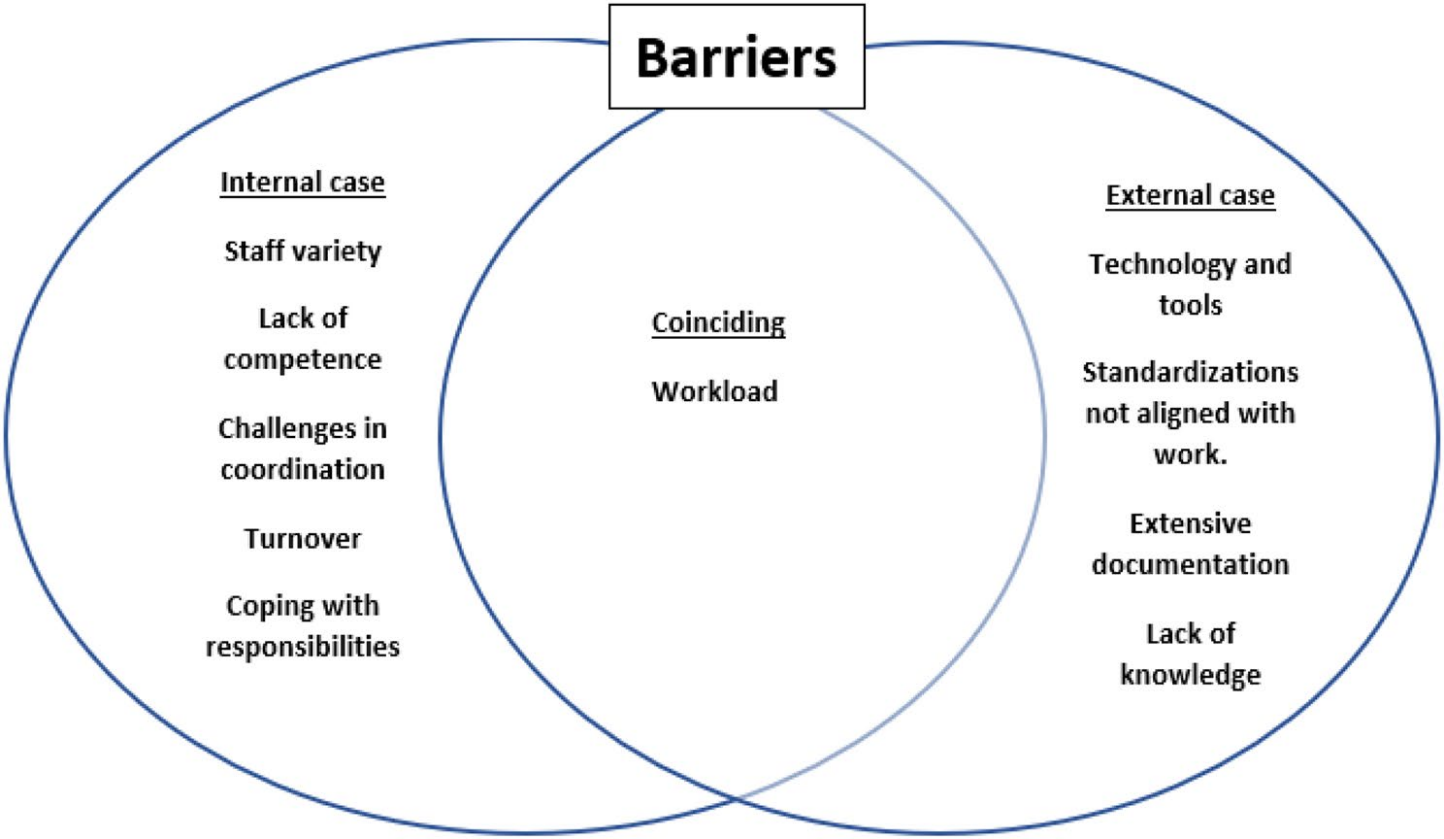
Barrier’s relatedness to externally and internally driven implementation processes

As displayed in Fig. 1, some 1st order codes were coincident for both the internal and external case, while others were of a more situation specific nature. As 1st order codes emerged inductively and were analysed separately within the two different cases and furthermore at different points in time, 1st order codes might therefore be named differently, like e.g., dedication and ownership (internal case) and engagement (external case) but with similar underlying meaning. The identified barriers and enablers of the two cases were in a second step put in a Venn diagram to explore and illustrate cross-case similarities and differences (see Figs. 2 and 3).

Table 2 illustrates the findings, in terms of different codes and the number of occurrences, according to RQ 1. The data structure model and the following result section describe successful and unsuccessful outcomes, focusing on most present occurrences within the dataset for each case to align with the first research question of identifying enablers and barriers of the implementation (Fig. 1; Table 2).

**Table 2:** Enablers and barriers with associated occurrences

Dimensions	Codes and occurrences
Enablers Internal	Continuous learning (22 occurrences) Systematic approach (22 occurrences) Simulation training (20 occurrences) Tools and technology (19 occurrences) Dedication and ownership (18 occurrences)
Enablers External	Knowledge sharing (35 occurrences) Coordination (33 occurrences) Engagement (33 occurrences) Technology and tools (31 occurrences) Perceived relevance (29 occurrences)
Barriers Internal	Staff variety (19 occurrences) Lack of appropriate competence (15 occurrences) Challenges in coordination (14 occurrences) Workload (11 occurrences) Turnover (11 occurrences) Coping with given responsibilities (11 occurrences)
Barriers External	Technology and tools (27 occurrences) Workload (25 occurrences) Standardizations not aligned with work (23 occurrences) Extensive documentation (21 occurrences) Lack of knowledge sharing (21 occurrences)

### Enablers

In the following different enablers, illustrated in Table 2; Fig. 1, will be described in more detail.

### Continuous learning, simulation training and knowledge sharing

The importance of continuous learning was emphasized in the internally driven implementation process. A continuous focus of learning was needed to ensure ownership of the QI programme, and for making new practices and knowledge an integrated part of the culture and everyday work. Leaders, through their decision to engage and to coordinate training, were found important for facilitating this continuity of focus. As such, having weekly simulation training was perceived more favourable than having yearly seminars for the staff. Continuous learning was also of importance for the external case, yet in a lesser degree due to having visits from researchers that provided reflexive spaces and learning at predefined points in time.

*“I’m so happy we engaged into this project, which gave us the results I expected. Yet, there is something that comes with this (the QI programme). We can’t stop with simulation training, as this needs to be of focus always.”* (Internal implementation process).

As the quote above states, simulation training was not a one-off activity, but needed to be put in a system of repeated and continuous exposure to be beneficial. However, when simulation training firstly was introduced to the staff, it was met with high levels of resistance. Simulation training made the staff feeling exposed and evaluated, which again made them feel insecure. As time went by and the staff got more familiar with simulation training, they changed their opinion of simulation training as they discovered its value, relevance, and the inherent flexible ability to illustrate everyday issues.

Simulation training was found a pillar for the internal case but was not a part of the external implementation process, which explains this difference of occurrences across cases. However, aspects of knowledge and learning were similarly found to be of highly importance for the externally driven implementation process. Knowledge sharing for the externally driven implementation process was mainly organized in inter-organizational workshops facilitated by the researchers. As such, these workshops acted as means to develop relations between leaders across different parts of the organizations and furthermore a way to learn from each other’s successes and mistakes when organizing for QI.

*“We know that A (home care department), which previously were a part of B (another home care department), has very good experience in initiating socio-professional evenings, which were very well attended. But, we (B) have struggled to make this happen. We are now planning to develop a culture for this (socio-professional evenings). We will therefore try to learn from their experiences. This is a way of using experiences from other organizations to make changes. Learning from the positive culture of other organizations”* (External case).

Other dimensions of knowledge sharing highlighted in the external case were having access to meeting arenas, to facilitate knowledge sharing across organizations, providing a direction of focus for the implementation. Furthermore, receiving feedback from different actors, both vertical (different levels) and horizontal (different departments) provided a more holistic understanding and knowledge of the contextual situation for the leaders.

### Perceived relevance

Willingness to put in necessary time and effort into the implementation process, relied on a perception of the outcomes to be relevant for their organization. The perceived relevance of the QI programme was found key for engagement and dedication in both cases. For the externally driven implementation it was not enough to just have motivated external researchers on visits to facilitate workshops, the motivation had to be present within the organization. Relevance in terms of quality further involved the development of a shared understanding of quality among healthcare professionals and their leaders. Hence, reflections over the meaning of quality were perceived as a valuable exercise.

*“Often, they (front-line staff) say: “We need more people at work”. Based on front-line staff, this is kind of the solution for everything. However, this is not the case. I mean, we can provide quality in a good way, even though our time is limited, by providing good quality in what we do. And by having good procedures”. (External case).*

Perceived relevance was also important for the internal implementation, even though this was raised in a lesser degree as the internal QI programme itself was specifically chosen by the leader for its relevance to their service.

### Dedication, ownership, and engagement

Dedication, ownership, and engagement was found enabling in both cases. Engagement was crucial for the ownership of new procedures and perspectives. For the external case, the informants highlighted a need for leaders to develop a firm ownership of the implementation, as explained in the following quote:

*“We had kind of decided this. We are a group that like to carry out things in clinical practice, not just making plans, but to actually realize them. I believe this is something we all are interested in. Therefore, it is important to make a plan, because it (the leader guide) needs to be anchored in management, and then we all have to work further to spread it (in practise). This is not something that can be done in 5 minutes, we must work with it (the leader guide) over time. And because this is something we have all agreed on, we will manage”. (External case).*

For the internal implementation process, ownership was associated with the adoption of new procedures. Learning new procedures and measures, made especially individuals with a lower level of formal education feel safer when making decisions, resulting in a reduced need for contacting nurses, physicians, and emergency departments to get advice and supervision. Furthermore, in situations where there existed a need for contacting other healthcare professions (like physicians and emergency departments), the new knowledge allowed healthcare professionals to concretize their information, making it easier for physicians and emergency departments to target their advice. This can be exemplified by the following quote.

*“They (health workers with lower formal education or no education) have evolved so much and have become so much more confident. They are the ones who have developed the most from this (the QI programme). And they have enjoyed it. And now they come back (to the home care central) and proudly stats: “I participated to a hospitalization”. This is so nice for us all to observe.* (Internal case).

### Technology and tools

Having access to appropriate tools, technology, and simulation equipment, were found enabling for implementation in both cases. It is important to notice, at this point, that technology and tools also were reported as a barrier. Meaning that technology and tools need to be accessible, easy to use, providing an overview, and easing information transfer to act as an enabler, if not, they may end up as a barrier. As such, the digital version of the leadership guide in the external case acted as an enabler providing accessibility to the learning resources, but also as a barrier if challenges in its use emerged.

One of the most important resources provided within the internal QI programme, was found to be the simulation equipment. Even though staff were reluctant to engage in simulations at first, they started to increasingly value this form of training as they got more familiar with simulation training. Findings showed that by only providing staff new tools (e.g., oximeters and blood pressure monitors) to not be sufficient to improve practises unless staff got properly trained in using them. Facilitating simulation training to ensure the correct use of new equipment was therefore enabling for internalization of new practices, as exemplified in the following quote.

*“We found it necessary to include certain procedures in the simulation sessions. We then discovered that some people measured blood pressure incorrectly, and that some measured temperature incorrectly. Respiration was not always measured correctly, and not always the pulse. Therefore, even though we have been providing training, we still need to repeat the training over and over”. (Internal case).*

### Systematic approach and coordination

A systematic approach provided continuity of the implementation process and furthermore acted as a support structure for both cases. For the internal case a systematic approach was needed to coordinate training of all staff in new procedures and measures.

*“Having a systematic plan for simulation training and lists of who is to be participating or not, has worked really well. And also, giving staff the responsibility for their own equipment, like the equipment bags, has also worked well”. (Internal case).*

While the internal case had to coordinate the full implementation process themselves, participants in the external case were given “homework” by the external facilitators to perform between each pre-set meeting. Systematization in terms of coordination of the homework was found highly enabling for the external case. Systematization and coordination of the implementation approach are therefore highly related aspects in this study. To provide ownership for participants in the external case, self-organization in the coordination of homework, like in defining of goals and the initiation of action plans, was found enabling as a way to align the implementation process to their specific context.

### Barriers

In the following different barriers, illustrated in Table 2; Fig. 1, are described in more detail.

### Workload, turnover, coping with given responsibilities, staff variety and challenges in coordination

There was consensus across cases on the influence of workload as a barrier for implementation. The daily and already busy workload for health care professionals meant that the implementation of new QI programmes put on extra strains and responsibilities for the participants and the organization. Coping with these extra responsibilities was found challenging for the participants even if they perceived the implementation to be highly valuable for quality.

*“They sent us to the course, which was nice. But suddenly I was responsible for all the simulation training, without being familiar with it. And I didn’t even want it….How was I to cope with this in daily practice, when I didn’t even have knowledge of it (simulation training)”* (Internal implementation).

The same holds for the externally driven implementation process where they were given “homework” to perform between workshops. This was perceived as extra work to be performed on top of their already busy schedule.

*“I remember well when it (the leader guide) was provided to us. And then I thought – how exiting. Really exciting. But then it (the leader guide) kind of got lost in everything else. The institutional leader quit her job at the same time, which also impacted us…However, to be able to have more focus on these things (quality improvement) would have been amazing”* (External implementation).

The quote above further points to another emerging code, turnover, which was perceived as an important barrier. For the internal case, the training of new staff in the QI programme practices was experienced as time consuming and the overall implementation process therefore got negatively affected by turnover, as described in the quote below. For the external case, turnover of key personnel during the implementation process was found to be a barrier as it was difficult to find replacements to take over as implementation agents, as described in the quote above. This reflects the importance of continued engagement of the implementation process to facilitate for the development of ownership and internalisation.

*“Barriers are typically turnover. And when training people in home care practices, there is a lot to teach them. It takes a really long time before you as a nurse or skilled health worker get hold of all the little things that we do here. So, this is difficult” (Internal case).*

Healthcare workers in primary care are a diverse group, with variations in formal education, experiences, and contextual knowledge. This means that the implementation process needed to be aligned to the receiver, something leaders at times found challenging due to the staff variety (competence, perspectives, experience, education), reflected in the quote below.

*“There are so many who should be seen and heard and all that. But I think this (QI programme) is important, especially for skilled health workers, it is important that they are lifted, and receive feedback on what they do well. Because there is a bit of rivalry between the professional nurses and the skilled health workers. Some professional nurses react when I praise them, because they feel this is only what should be expected. While others…It is difficult to applaud too much because you never know how it is received”* (Internal case).

### Technology and Tools

Technology and tools were found to possess a dual role as both enabler and barrier. When technology and tools were acting as a barrier it was caused by limited accessibility, compatibility, and output. The leadership guide in the externally driven implementation process was provided to the participants in both a paper version and as a digital website version. Most participants preferred the digital version due to accessibility. However, some elements of the digital version were perceived challenging by the participants, like the storing of the results, and as such a barrier for progress with the “homework”.

### Lack of appropriate competence and knowledge sharing

The large variety in terms of formal education and contextual experiences meant that some employees felt that they were missing the appropriate competence to perform some specific practices. Like in the internal case where some of the simulation facilitators were unfamiliar with what they were expected to learn to their colleagues. As such, some facilitators needed more knowledge and training themselves before having to initiate training for others.

*Some of the professional content has been very difficult, since I’ve not previously worked with this stuff. Among this, the use of the elevator. We have worked with a scenario of falls. I know falls very well, but I cannot use the elevator. And suddenly I’m supposed to teach the others in how to use the elevator”.* (Internal case)

Knowledge sharing was also found to possess a dual role as both enabler and barrier, which reflects the importance of knowledge sharing for implementation. For the external case, lack of knowledge sharing was related to a lack of suitable arenas for knowledge sharing. Formalized meeting arenas were limited, which often resulted in a need to share knowledge, and to receive feedback, of more or less everything within the same meeting. As such, raised topics were only dealt with superficially.

*“We have discussed this a bit between us. We want to have a forum. Yes, a forum for adverse events where we can air adverse events on a general basis”* (External case*).*

### Standardizations not aligned with work and extensive documentation

Standardizations were perceived as counterproductive for quality improvement if the standardizations and routines were not properly aligned with daily work. The informants also reported that the number of standardizations, guidelines, and routines to be implemented made them less compliant to keep up with them all.

*“I’m much less compliant to standardizations from the government, and the municipality, of what is of importance at the moment. This has nothing to do with quality, it is about logistics, so let us instead focus on quality.”* (External case).

The same also holds for documentation. If standardizations, guidelines, and procedures required extensive documentation, then health care professionals were more reluctant to engage. A consistent problem in home care services was the use of computers. Healthcare professionals only had access to I Pads when visiting patients, meaning that they had to wait until they were back at the home care central to perform reporting and documentation. Another problem was the limited number of computers available at the home care central, meaning that they sometimes had to que up for reporting and documenting. The number of different software to enter for reporting and documenting, with corresponding passwords, was also perceived a barrier for compliance.

*“The staff do not sit in front of a computer. It is very difficult to get them to use the computer, using their e-mail, and all this. It is a big challenge because they are not using the computer unless when they are writing up the report”* (External case).

### Cross-case analysis

In accordance with RQ2 the results described above were structured in terms of coinciding and context specific factors, for which the visual result provides us some new understanding, see Figs. 2 and 3. It is important to notice that all factors influenced the implementation process, and each other, in rich and complex ways. For enablers, most factors were found coinciding across the cases. This means that the identified coinciding factors, were important for succeeding with healthcare implementation and should be of focus for leaders, facilitators, and implementation agents.

However, when identified barriers were structured across cases in a similar diagram, an opposite pattern surfaced. Workload was the only coincident factor across the cases, and the other factors were found more context specific. This pattern provides the understanding that workload is a common and important barrier, that needs to be of focus in all implementation processes. Yet, to circumvent barriers, contextual knowledge of the implementation setting, and furthermore characteristics of the QI programme, are necessary to understand contextual barriers for implementation. By having contextual knowledge, leaders, facilitators, and implementation agents can work to align the implementation process to the situational context, like available resources, level of competence, turnover, staff variety, technology, and the appropriate level of documentation and standardization.

## Discussion

We performed a qualitative study in nursing homes and home care services and identified enablers and barriers for externally and internally driven QI implementation processes. We found that technology and tools, ownership, and learning were coinciding enablers, while workload was a common barrier across the implementations processes. We also identified several barriers and enablers that differed between the externally and internally driven implementation processes. In the following we discuss these and relate them to previous research and especially the conceptual and layered framework of Lau et al. [11] focusing on intervention characteristics, external, organisational, and professional factors to understand success and failure in QI implementation processes. The frameworks by Lau et al. [32] and Damschroder et al. [33] have a different focus of study and the relatedness to these frameworks were therefore only identified after the indictive data analysis was completed.

### Conceptual framework for enablers and barriers of healthcare implementations

**Fig. 4:**
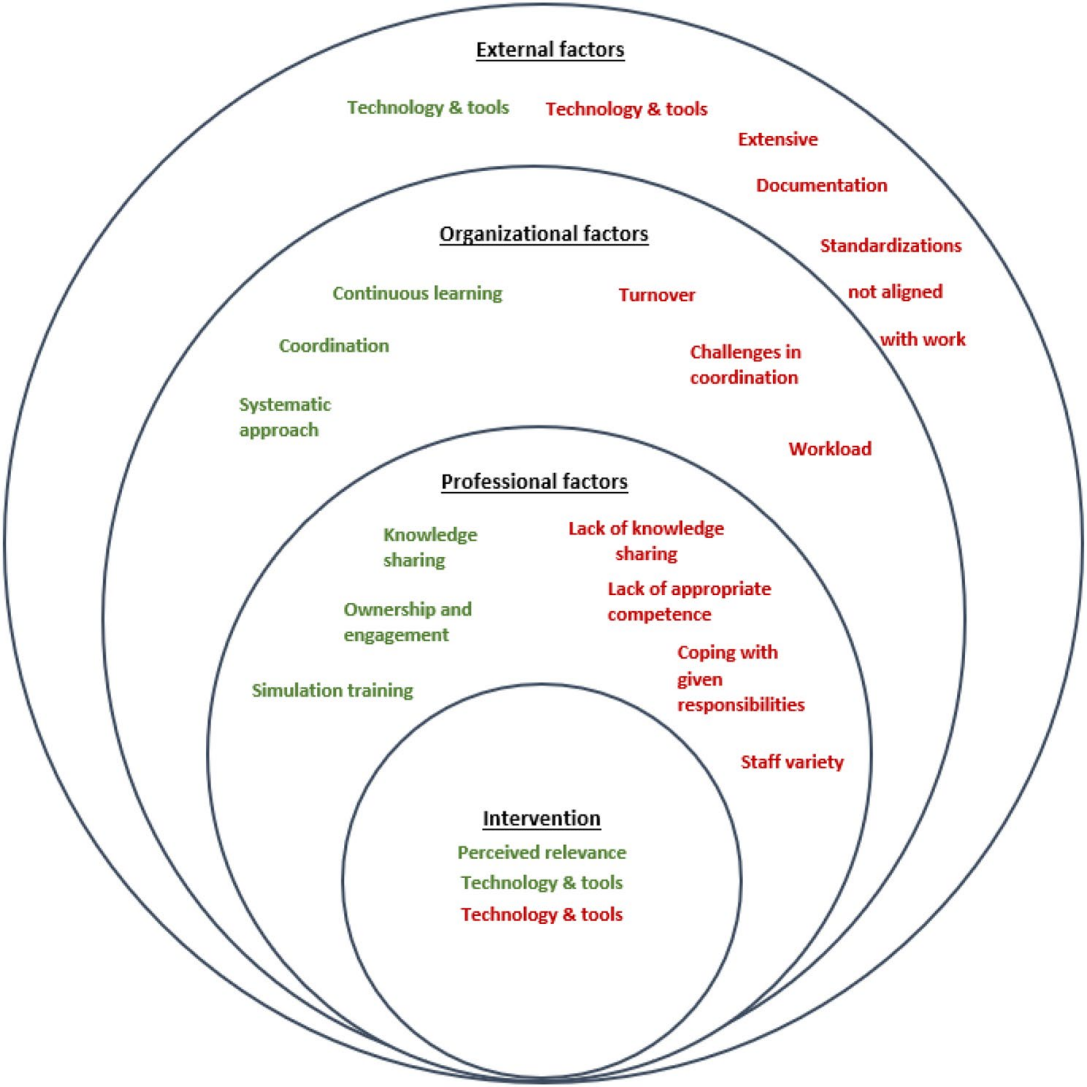
Enablers and barriers from both cases in relation to the intervention, and professional, organizational, and external factors. Green text refers to enablers and red text refers to barriers. Adapted from Lau et al. (2015)

The findings illustrate diverse enablers and barriers for externally driven and internally driven implementations. Exploring the results from this study in relation to the framework by Lau et al. [11], the following categorization depicted in Fig. 4 can be developed. Each level (external, organisational, professional, intervention) influences the others, meaning that the different levels are interdependent elements of implementation processes and need to be understood as a contextualized entirety. Each level will be described in more detail in the following.

### Factors concerning the intervention/QI programme

Factors associated with the nature of the intervention/ QI programme itself refer to the identified enabling factor of perceived relevance. Relevance aligns with what Lau et al. [11] identified in their review on the importance of relevance, clarity, and practicality, all pointing to a need for the QI programme to provide benefits for practice, economy, patient safety, and efficiency. Efforts to make the QI programme perceived as relevant, relied on a fit between the situational context and the QI programme, thereby highlighting a need for user involvement during QI programme development. This finding is echoed in Cresswell et al. [7] where an alignment to the organizational context is not perceived as satisfactory unless the QI programme is aligned with clinical needs as well. Lau et al. [11] furthermore points to a need for leaders and implementation agents to clearly state the relevance of the QI programme in early phases of the implementation process, to provide willingness of staff to engage. This was also present in the external case where the researchers had to highlight the relevance of the QI programme for the participants to raise engagement and ownership [34, 35]. Damschroder et al. [14] and Ree et al. [35] reports adaptability of the QI programme as a key factor for ensuring fit to the situational context. This is also reflected in our study where outcomes of the QI programme, in terms of quality, needed to be adapted to their specific context to be classified as relevant. Granja et al. [3] in a literature review found perceived quality for healthcare to be the most mentioned category for success, relating quality to contributions for professional performance standards and clinical practice.

Technologies (digital version of the leadership guide in the external case) and tools (clinical equipment in the internal case) were also part of the QI programme itself and could introduce both positive (accessibility and improved practices) and negative impact (software challenges and lack of appropriate competence) to the implementation process, as described in the result section.

### Professional factors

Moving to the next circle of professional factors, which refers to influence, competence, and self-efficacy [11]. Professional factors encompass several findings within this study; enabling factors include ownership and engagement, simulation training and knowledge sharing, and barriers refer to a lack of appropriate competence and knowledge sharing, coping with given responsibilities, and staff variety. The marked role of knowledge sharing is evident through its position, as both an enabler and, if missing, a barrier. Knowledge sharing is essential for the dissemination of information, knowledge, and experience in both formal and informal channels throughout the implementation process. Furthermore, a lack of appropriate knowledge was also found hindering. This is reflected in the internal case where the staff variety made knowledge transfer more difficult, as they held different levels of competence and experience and thus required different resources for learning and training. This corresponds with Lau et al. [11] stating that adequate competence act as a facilitator for implementation success. Furthermore, Cresswell et al. [7] describe actors in possession of appropriate knowledge to be more positive towards new technology and services, than actors without appropriate training. The authors also describe a need for tailoring the training and to make it close to practise, which provides understanding to the importance of simulation training for the internal case [7].

Ownership was found of influential in both the internal and external implementation process. This corresponds with Lau et al. [11] who emphasize motivation and attitudes as enabling for change. For the external case, having the opportunity to adapt the implementation process to their setting was key for developing motivation. This issue of self-organization was not raised in the internal case as they by definition were responsible for the organization of the implementation. Greenhalgh et al. [10] describe ownership and adoption as a process, needing time to develop. Facilitators for this process were described to be appropriate knowledge of the output of the innovation/ QI programme (perceived relevance and appropriate competence), continuous access to information (continuous learning), ability for contextual adaptation, and to receive adequate feedback (knowledge sharing). These findings from Greenhalgh et al. [10] illustrate interdependencies of their findings and furthermore the findings of our study, pointing to the need for keeping a holistic perspective of barriers and enablers.

### Organizational factors

Continuity in learning enabled implementation, as in the internal case where continuous learning ensured ownership by repeated focus over time until new knowledge and procedures were internalized in form of routines and thought worlds. Other aspects of continuity, like in the external case where leadership turnover resulted in a disruption of the implementation process, also acted as a barrier for the implementation process. This echoes Damschroder et al. [14] who described continuity and stability of staff as facilitative for implementation success. Leadership is also highlighted in Lau et al. [11] study, as means to identify champions, drive change, and for the communicating of objectives for the implementation process. The authors further report that early engagement by leaders as valuable for the adoption of the QI programme. This understanding is echoed in this study in relation to leaders’ dedication and engagement to the implementation process.

Another factor that acted as both an enabler and a barrier was coordination. Well organized and structured plans for the implementation process were found facilitative. Allowing participants autonomy and room for self-organization were emphasized as a way of adapting the implementation process to front-line work in our study, similarly to Cresswell et al. [7]. This refers to what is described by Greenhalgh et al. [10] as fuzzy boundaries, where innovations in services organizations, like healthcare, includes a hard core of fundamental elements that need to be unaltered to maintain the purpose of the innovation, and a soft periphery providing adaptation to the context [8, 12, 27]. This is further echoed in this study where QI programmes involved a fundamental hard core (different measurements and procedures to perform/leadership guide with assignments) and a soft periphery (how, when, where, and with whom, learning and homework are performed).

However, coordination also acted as a barrier, put forward due to the complexity in care and the huge variety in training and education among staff. This understanding is also mirrored in the description by Lau et al. [11] on how skill mix issues may impede coordination of responsibilities and roles. Related to coordination, was also the enabler of having a systematic approach. Systematization of the implementation process was perceived as a support structure, as it eased the overview of the training process and furthermore provided an ability to monitor progress. This means that the flexibility to adapt and translate the QI programme, described facilitative in the above, needs to be accompanied with some support structures, like infrastructure and planning to ease the implementation process [7].

Workload was heavily reported as a barrier in this research, where limited time and effort to engage into the implementation resulted in a lack of ownership and engagement. Workload as a barrier for implementation is previously described in various research. Granja et al. [3] found workload to be overrepresented as a barrier within their systematic review, and Carlfjord et al. [2] describe workload to reduce the ability to engage into the implementation process. The implementation of new QI programmes therefore needs to align adoption efforts to workload, or else the implementation process may end up as a burden for healthcare workers already in a pressed and hectic work situation [36]. Bates and Singh [1] describe workarounds, for time saving purposes, as outcomes if the QI programme and implementation process are not aligned with workload.

### External factors

Nursing homes and home care services are the responsibilities of Norwegian municipalities. However, even if this structure allows for local decisions and prioritizations concerning quality improvement, the municipalities still need to align to national regulations, guidelines, and external factors. External factors found to influence the implementation were technology and tools, standardizations not aligned with work, and extensive documentation. Technology and tools therefore acted as both an enabler and a barrier for the implementation cases in this study. This is in line with Greenhalgh et al. [10] arguing that when tools are perceived as easy to use, the potential for successful implementation increases. Furthermore, appropriate, and useful knowledge for how to use technology and tools need to be present, findings which are echoed in Cresswell et al. [7].

When not perceived as accessible and useful for daily practise, technology and tools introduced a lack of motivation and workflow, thereby reducing the perceived incentives of engagement. Disruption of workflow was a significant finding in Granja et al. [3] due to a gap between the technology and the context. As a barrier for implementation, technology and tools are related to extensive documentation, referring to a lack of compliance to demands of documentation due to insufficient compatibility in technological systems, increased workload, limited resources, and poor functionality. This is furthermore mirrored in Lau et al. [11], stating that infrastructure, technology advances, and a lack of clear incentives are important external factors of implementation. Standardization and documentation, not aligned with work, are factors mostly initiated and decided upon externally, like from the specific QI programme itself, and from regulations and guidelines from the national level. However, even if decided outside of the organization, it is to be performed by individuals and teams within the organization. This means that some room for adaptability to context need to be present, if not they may act as a bottleneck for efficiency.

The Norwegian government has initiated a regulation stating the need for healthcare leaders in nursing homes and homecare services to ensure continuous quality improvement within their respective organizations. As such, there are formalised requirements for primary care leaders to take on different quality improvement interventions. However, which type of interventions to implement will often be up to the primary care leaders to decide upon, except for national programs where all units are to engage. The guidelines for which type of approach to use for the implementation process are also formed with flexibility for leaders to decide, based on what is most appropriate for their organization. Awareness of enablers and barriers for different approaches can potentially be of support for leaders in their quality improvement work. Furthermore, our findings display the benefit of providing flexibility for local adjustment of the intervention and implementation process which can inform policy makers to provide a room for adjustments in national programs.

### Strengths and limitations

This study has some limitations. First, as in all qualitative research the findings from this study are not transferable. Second, it may be considered a limitation that the internal and the external case implemented different QI programmes. However, a principle for the overall study was to allow the internal case to choose their own QI programme and furthermore to implement the QI programme on their own without any impact from the researchers. Third, it is relevant to mention that the internal case only included home care services, while the external case included both nursing homes and home care services. Forth, only interviews of leaders and professional nurses were included as empirical data for this study to explore the perspective of the implementation agents and to make the empirical foundation more similar across the cases. Other findings may therefore have emerged if the dataset also incorporated employee interviews and observation notes. New studies should therefore seek to perform similar studies informed by both leaders and employees. Fifth, the data collection in the external case included both individual and focus group interviews, while the internal case included only individual interviews. For the external case, the two individual interviews were chosen due to the geographical distance of this nursing home. Furthermore, the two leaders were not located in the same unit so individual interviews were found more convenient. The choice of using individual or focus group interviews have both pros and cons, where focus group interviews allow for more discussion and as such a way to generate more ideas, while individual interviews provide psychological safety as what is reported is only between the researcher and the informant [37, 38]. An additional factor to balance was the use of the leader’s time to conduct the interview. The interviews were therefore performed at the leader’s location which made it necessary to allow for both focus group interviews and individual interviews [39].

The major strength for this study is the comprehensive dataset, including several municipalities and institutions. The data was collected over time, providing credibility to the study. Additionally, the focus of leaders in both cases provides the ability for cross-comparison of the different implementation processes.

## Conclusion

The aim for this study was to explore enablers and barriers of two different implementation processes of QI programmes in nursing homes and home care services, where the implementation in one case entailed an externally driven process, while the second case entailed an internally driven implementation process. As such this study theoretically contributes to calls for research on different contextual settings of healthcare implementations and further to calls for implementation studies to take place in primary care settings.

This study shows that workload acted as the main barrier for implementation in both externally and internally driven implementation processes. Based on the current state of primary care with burnout, stress, and lack of qualified healthcare professionals causing a mismatch of demands and capacity in healthcare services, implementation of QI programmes needs to be aligned with their everyday work to not be perceived an extra burden [36].

Other barriers and enablers in need of extra focus in implementation processes are factors that acted as both barriers and enablers, like technology and tools, continuity in learning and staff (turnover), and coordination. Tightly monitoring these factors throughout the implementation process may provide a positive impact.

Dedication, engagement, and ownership are other factors which need to be emphasized. These enablers were raised in both cases. However, the influence of other enablers and barriers to facilitate or hamper the development of dedication, engagement and ownership leaves these factors of importance for implementation processes.

Future studies should seek to explore similar phenomena in different countries and at different parts of healthcare services, to develop an understanding of the findings in different contexts. Furthermore, future studies should also explore implementation of different QI programmes and compare different types of implementation processes in terms of timespan, level of change introduced by the QI programme, and alternative ways of facilitating the implementation process, like having mixed externally and internally processes, as well as front-line initiated and government-initiated QI programmes.

### Electronic supplementary material

Below is the link to the electronic supplementary material.

Supplementary Material 1

Supplementary Material 2

Supplementary Material 3

## Data Availability

The datasets used and analysed during this study are available from the corresponding author on reasonable request.

## Abbreviations

QI: Quality improvement
SAFE LEAD: Improving Quality and Safety in Primary Care – Implementing a Leadership Intervention in Nursing Homes and Homecare
SHARE: Centre for Resilience in Healthcare, Faculty of Health Sciences
USHT: Centre for Development of Institutional and Home Care Services
