# Supplementary material for: Barriers and enablers for externally and internally driven implementation processes in healthcare: a qualitative cross-case study
Source: BMC Health Serv Res. 2024 Apr 25;24:528. doi: 10.1186/s12913-024-10985-2 (PMC11046894; doi:10.1186/s12913-024-10985-2)
Supplement: Supplementary file 1 — Supplementary Material 1 [file 12913_2024_10985_MOESM1_ESM.pdf]

External case. Leader interviews. Before implementation

Background data (age, education, work experience, position)

Structure:

- What is your understanding of quality and patient safety?
- How does your unit work with quality improvement? And how is this work organized (roles, responsibilities)?
- Which types of forums exist?
- Are there objectives, strategies and plans for quality improvement?
- Have you access to tools, guidelines, interventions, and methods for quality improvement work? Which ones? Do you miss any tools?
- Do you have experience with patient involvement in quality improvement work? If so, how is the patients and next-of-kin involved?
- How do you act on adverse events (reporting, information to next-of-kin, handling the involved staff, procedure, competence for analysing adverse events)?

Culture and engagement:

- How can a shared understanding of quality and patient safety be developed?
- What factors hinder quality improvement work?
- What factors facilitates quality improvement work?
- Can you describe a situation where your work on quality improvement provided a good outcome?
- How do you work with quality improvement?
- How do you work to increase engagement of quality improvement work?
- How do you work to ensure patient centred care and positive patient experiences?

Competence:

- How do you facilitate for competence building?
- How do you ensure necessary time to work on quality improvement work?
- Who takes the initiative for initiating changes related to quality improvement in the unit?
- Do you experience previous quality improvement implementations as valuable for your work situation?
- How do you perform local adjustments of national guidelines?
- How do you perceive leaders (level above) facilitates for implementations of quality improvement programmes?

Coordination:

- What type of networks can you use in the work on quality improvement?
- Do you interact with other experts/professions in your work on quality improvement?

- How do you collaborate with the hospital in the discharge of patients to nursing homes/home care services?

Physical and technological environment:

- How do you perceive present information systems facilitates for quality and safety work?
- How do you perceive the physical environment to facilitate for quality and safety work?

Outer context:

- How do you work to implement national guidelines in your organization?
- In what ways have you experienced external demands and regulation to facilitate or hinder your work on quality improvement?

Anything else you would like to inform us about?

External case. Leader interviews. After implementation.

1. Background data (age, education, work experience, position)
2. What expectations did you have beforehand about implementing the leadership guide?
3. Generally, how have you experienced the implementation of the leadership guide?
4. Have you used the leadership guide as you planned for?
5. How have you experienced it to be part of the implementation process?
6. Have you used the leadership guide in your unit/organization? Who participated and in what form?
7. Have you succeeded/not succeeded with the implementation in this unit/organization? What do you perceive to be the most important factors for the implementation process in your organization?
8. Have you increased your knowledge/competence of quality improvement work by participating in this implementation process? Why and what type of competence?
9. Has the leadership guide contributed positively to quality improvement work?
10. Which of the 7 included challenges in the leadership guide have you as a leader focused on in the implementation process?
11. What part of the leadership guide has been most valuable for your work on quality improvement?
12. Do you experience patient involvement to be facilitative for qualitative improvement work?
13. What were the barriers for this implementation process?
14. How can we improve the leadership guide?
15. How can we improve the implementation process?
16. How do you perceive the relevance of the introduction and training included of this implementation process?
17. Would you recommend other to take part in t implementation of the leadership guide?
18. If you could mention, in one word, what was experienced as the biggest enabler and the biggest barrier for the implementation process?
19. Is there anything else you would like to inform us about?
