# Supplementary material for: Barriers and enablers for externally and internally driven implementation processes in healthcare: a qualitative cross-case study
Source: BMC Health Serv Res. 2024 Apr 25;24:528. doi: 10.1186/s12913-024-10985-2 (PMC11046894; doi:10.1186/s12913-024-10985-2)
Supplement: Supplementary file 2 — Supplementary Material 2 [file 12913_2024_10985_MOESM2_ESM.pdf]

Internal case.
Interview guide leaders and Professional nurses.
Phase 3.

1. Brief introduction of yourself (age, education, work experience) and whether you previously have been participating in interviews or observation?
2. How have you been involved in the implementation of the QI programme?
3. Can you describe your experiences of the QI implementation?
    a. How have you experienced the initiation of the implementation process?
    b. What parts of the QI implementation have you taking part in?
    c. How would you evaluate the different parts of the QI programme (compendium, teaching, e-learning module, simulation, technology and tools, equipment backpacks, ISBAR)?
4. How do you perceive the QI implementation, what has worked well and what has not worked well?
    a. What are the enabling factors and what are the barriers?
    b. How do staff contribute to change/do not contribute to change?
    c. How do you as a leader contribute/ not contributing to change?
    d. How do organizational factors contribute/not contribute to change (new employees, temporary staff, workload, feedback from staff)?
5. How do you evaluate the results of the QI implementation?
    a. Change of practises?
        - Are the routines for clinical assessments of patients changed?
        - Are there changes in the way staff observes and evaluates deteriorating patients? If so, in what way?
    b. What is the impact of the QI implementation for healthcare personnel i home care services?
        - Have the QI programme changed the way you/they think around clinical observation and evaluation of patients? If so how and where is this visible (expressed)?
        - How do you think the QI programme have contributed to competence (theoretical and practical)?
        - Do you experience your staff to have the appropriate competence for identifying patients in deterioration? Why/why not?
    b. How do you as a leader evaluate the impact of this QI implementation process for patients in home care?
        - Can you describe situations of deteriorating patients and how this have been handled? Concrete examples.
    c. What is the overall impact of the QI implementation process for this department/zone/home care service?

Anything else you would like to inform us about?
